# Diagnostic Value of Circulating microRNAs in Hepatitis B Virus-Related Hepatocellular Carcinoma: A Systematic Review and Meta-Analysis

**DOI:** 10.7150/jca.32833

**Published:** 2019-08-20

**Authors:** Xuehang Jin, Changzhou Cai, Yunqing Qiu

**Affiliations:** 1 State Key Laboratory for Diagnosis and Treatment of Infectious Disease, Collaborative Innovation Center for Diagnosis and Treatment of Infectious Diseases, Zhejiang Provincial Key Laboratory for Drug Clinical Research and Evaluation, The First Affiliated Hospital, Zhejiang University, 79 QingChun Road, Hangzhou, Zhejiang 310000, People's Republic of China.; 2 Department of Gastroenterogy, The First Affiliated Hospital of Zhejiang University School of Medicine, Hangzhou 310003, China.

**Keywords:** hepatocellular carcinoma, hepatitis B virus, circulating microRNA, biomarkers

## Abstract

Chronic hepatitis B virus (CHB) infection is the leading cause of hepatocellular carcinoma (HCC). As it is difficult to diagnose the early-stage hepatocellular carcinoma using the existing approaches, better biomarkers are urgently needed and may improve the patients' prognoses. MicroRNAs are the most studied liquid biopsy biomarkers and multiple studies have demonstrated the significant diagnostic value of miRNA in HBV-related hepatocellular carcinoma. In this meta-analysis, we collected 25 studies from 15 researches that included a total of 2290 HBV-related HCC patients and 1551 HBV patients without HCC. The pooled sensitivity, specificity, PLR, NLR, DOR and AUC were 0.84 (95% CI: 0.79-0.88), 0.75 (95% CI: 0.69-0.81), 3.42 (95% CI: 2.68-4.35), 0.21 (95% CI: 0.16-0.29), 15.99 (95% CI: 9.89-25.83) and 0.87 (95% CI: 0.83-0.89), respectively. Subgroup analysis indicated that multiple microRNAs, downregulated miRNAs assays, serum type and big sample size had much better accuracy and miR-125b especially, showed a significant diagnostic value. In addition, there is no obvious dignostic difference for HCC from both chronic hepatitis B and liver cirrhosis (LC). Publication bias was not found and Fagan's Nomogram showed valuable clinical utility. In conclusion, circulating microRNAs, particularly the miR-125b, may serve as promising biomarkers for the early diagnosis of HBV-related HCC. However, larger and more rigorous studies are needed to confirm our conclusions.

## Introduction

Hepatocellular carcinoma (HCC) is predicted to be the fifth most commonly diagnosed malignancy and the fourth leading cause of cancer-related death in 2018, with an incidence of 841,000 new cases and 782,000 deaths per year 1. The chronic viral infection is the main cause and it has been reported that Hepatitis B Virus (HBV) infection causes approximately 80% of HCC. As a result, the early diagnosis of HCC from HBV-related hepatic diseases is obviously crucial. At present, the detection of HCC is mainly relied on the radiological imaging studies, including ultrasonography, computed tomography (CT) and magnetic resonance imaging (MRI). However, owing to the coexistence of inflammation and cirrhosis, the detection of early stage HCC is difficult and sometimes need liver biopsy for further verifying 2. Alpha-Fetoprotein (AFP), the most commonly used serum biomarker, has the poor sensitivity and specificity of 41% to 65% and 80% to 94%, respectively, and can only detect the one-third of early HCC patients at the threshold level of 20 ng/mL 3-5. Above all, advanced strategies for early detection of HCC from liver cirrhosis (LC)and chronic hepatitis B (CHB) with high accuracy are urgently needed.

MicroRNAs, a class of non-coding single-stranded RNAs (19 to 25 nucleotides long) , are the translational inhibitors of their target mRNAs by means of binding to the complementary sequences in the 3′- untranslated region (UTR) and result in the repression of relevant protein expression 6, 7. It has been confirmed that microRNAs play a crucial role in all kinds of cellular processes, including proliferation, differentiation, metabolism and death 8, 9. Most of all, various of studies have demonstrated the link between the deregulation of microRNAs and tumorigenesis. Further more, many researches about the specific microRNA have shown the ideal accuracy in the diagnosis of cancer, including hepatocellular cacinoma. One of the recent studies reported by chen et al. 10 measured the plasma-based miR-125b and concluded a significant accuracy in discriminating the HCC with CHB or LC, with a sensitivity of 93.8%/ 89.1% and specificity of 85.7%/88.1%, respectively. In addition, microRNA panels are also being identified and Zhou et al. 11, who measured a 7-microRNAs panel, also got a satisfying diagnostic achievement. With the advantages of its high stability in circulation, easily detection using PCR-based methods and better diagnostic value, microRNAs are becoming a novel and promising biomarker for early detection of HCC 9, 12.

Several meta-analyses have investigated the diagnostic value of circulating microRNAs for hepatocellular carcinoma, showing the significant value of serum/plasma microRNAs in detecting the early-stage HCC 13-16. However, no one made a point of the HBV-associated HCC. For making a further step to evaluate the risk of HCC in patients with HBV chronic infection, we conducted a meta-analysis of all suitable researches to explore the ability of circulating microRNAs as potential biomarkers to detect the HBV-related HCC from CHB and HBV-related LC patients.

## Materials and Methods

### Search strategy and literature selection

This meta-analysis was conducted according to guidelines for the diagnostic meta-analysis. We systematically searched the literatures in PubMed, PMC, Web of Science, Embase and the Cochrane library without language restriction and used the following retrieval terms: “liver cancer” or “hepatocellular carcinoma” or “HCC” and “microRNA” or “miRNA” and “hepatitis b virus” or “HBV” or “chronic hepatitis” or “liver cirrhosis”. The last search was carried out on 30/11/2018.

### Inclusion and exclusion criteria

Literatures that included in our meta-analysis met the following criteria : (1) all of the involved patients and controls were HBV-related; (2) the microRNAs for HBV-related HCC diagnosis were detected from serum or plasma. (3) concern the use of relevant data , such as specificity, sensitivity, group size or other sufficient information to construct a diagnostic 2×2 table. On the other side, exclusion standards were : (1) literatures were case reports, reviews, letters or comments; (2) duplicated information; and (3) the obtained microRNAs were from liver tissues, urine, cell lines or animals.

### Data collection and study assessment

Two investigators selected and screened the relevant studies independently based on the title and abstract, and the full text, which was reviewed for further assessment if the study was collected by either of the investigators. We obtained the following datas from the each eligible researches : the first author's name, publication year, ethnicity, miRNA type, the number of HCC/CHB/LC patients and the revelant statistical index. The quality of included studies were assessed independently by two investigators using diagnostic accuracy studies-2 (QUADAS-2) criteria 17 , and disagreement was resolved by consulting to each other and reached a consensus.

### Statistical analysis

The number of true positives, false positives, false negatives, true negatives in patients from each study was extracted. The heterogeneity was evaluated by I2 statistic. The random effects model was conducted if the I2 value was more than 50%. The potential sources of heterogeneity were explored by threshold effect analysis, regression analysis and further subgroup analysis. We then summarized the pooled sensitivity (SEN), the pooled specificity (SPE), the pooled positive likelihood ratio (PLR), the pooled negative likelihood ratio (NLR), the diagnostic odds ratio (DOR). Besides, we generated the summary receiver-operating characteristics (SROC) curve and calculated the area under the SROC curve (AUC) for both overall and the subgroup analysis. Apart from that, a Fagan's Nomogram was generated for verifying clinical utility. Finally, a Deeks' funnel plot used for detecting publication bias was constructed, and P < 0.10 indicated publication bias. All of these were performed by STATA version 12.

## Results

### Study selection and literature characteristics

**Figure 1:**
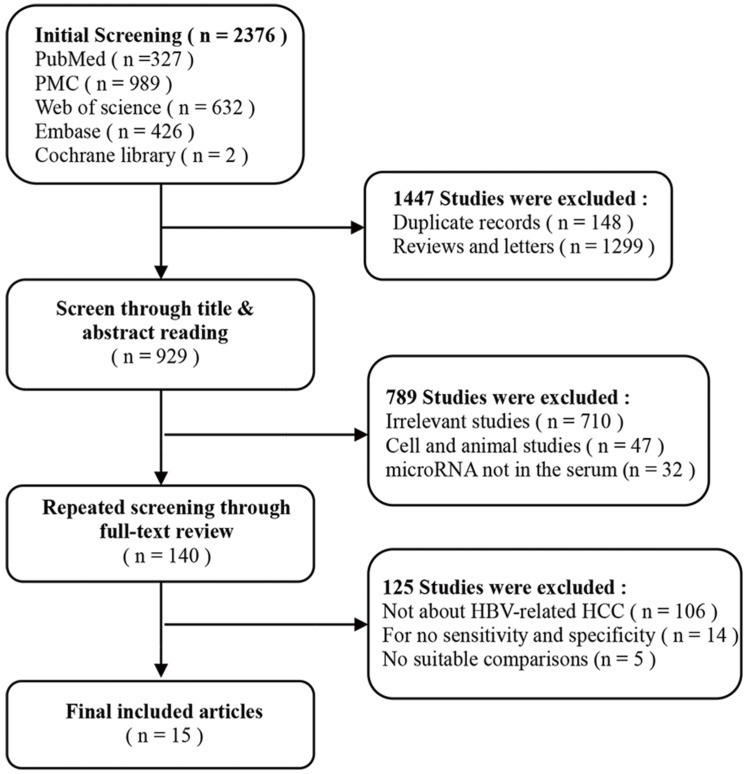
The flow chart of this systematic review and meta-analysis to identify inclusion studies.

**Figure 2:**
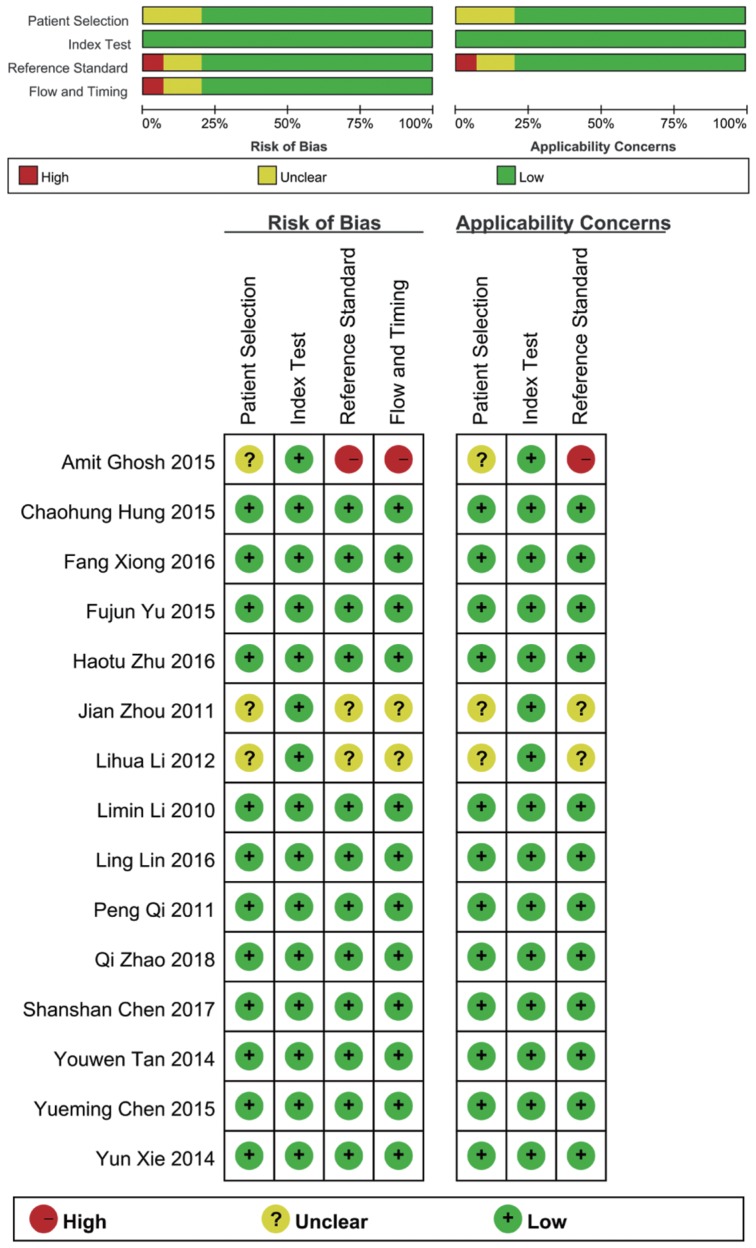
Overall methodology quality assessment of included articles using the QUADAS criteria.

According to the literature retrieval strategy, a total of 2376 articles were acquired, of which, 327 were from Pubmed, 989 were from PMC, 426 were from Embase, 632 were from Web of Science and 2 were from Cochrane. After that, 148 duplicates, 1299 reviews and letters, 47 animal researches, 710 irrelevant studies, 106 not about HBV-related HCC, 14 articles without available diagnostic information and 37 other articles (Figure 1) were removed. Eventually, 25 studies from 15 articles 10, 11, 18-30 were included in our meta-analysis. The characteristics of the 25 studies were shown (Table 1). All together, a total of 2290 HBV-related HCC patients and 1551 HBV patients without HCC were included. In all, 20 miRNA studies concerned a single miRNA, and 5 studies focused on multiple miRNAs. Apart from that, quantitative real-time reverse transcription-PCR (qRT-PCR) was used to measure the expression of miRNAs from 21 serum specimens and 4 plasma specimens. The methodological quality assessments of the included articles according to the Quality Assessment of Diagnostic Accuracy Studies (QUADAS) were shown in a bar graph (Figure 2).

### Threshold effect

By matching the ROC curve, using the logarithm of sensitivity and the logarithm of (1 - specificity) to calculate the Spearman correlation coefficient, the threshold effect was assessed. The results showed the shape of the ROC curve not like arm and shoulder shaped distribution. The Spearman correlation coefficient in total across the 15 studies was 0.000 (P = 0.999), which indicated no threshold effect.

### Diagnostic value of circulating microRNAs in HBV-related HCC patients

**Figure 3:**
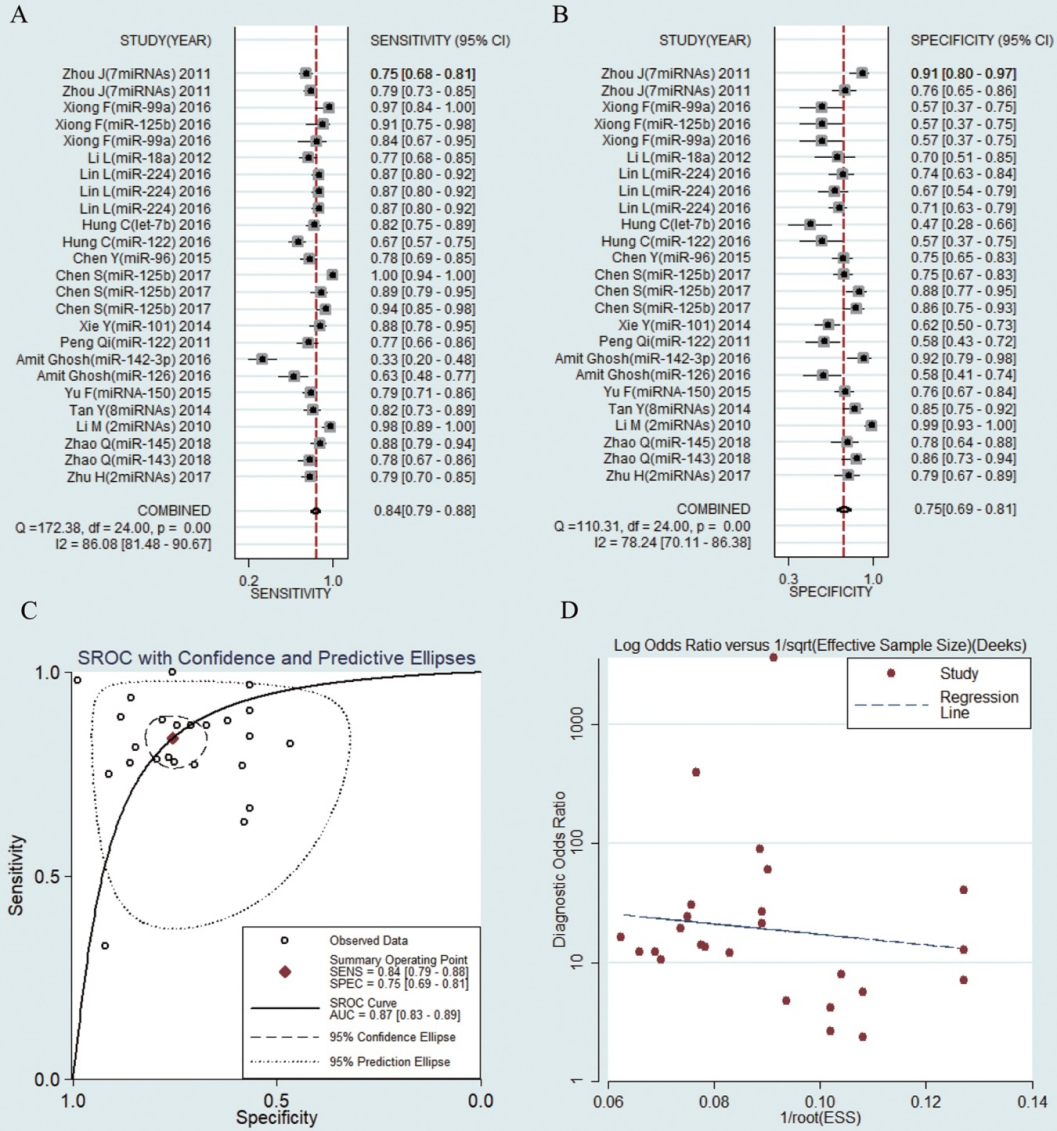
Forest plots of sensitivity, specificity, area under the curve (AUC) and funnel plot of circulating miRNAs for diagnosing HBV-related HCC among 25 studies. (A) Sensitivity; (B) Specificity; (C) AUC; (D) Funnel plot.

The sensitivities and specificities of the 25 microRNAs in the peripheral blood circulation of HBV-related HCC patients were analyzed by using forest plots. Significant heterogeneity existed among the studies from the data of diagnostic odds ratio (DOR) (I2 = 100%) (Fig. 3), and therefore, the random effects model was selected in our meta-analysis. The pooled results were displayed as follow: sensitivity, 0.84 (95% CI: 0.79-0.88) (Fig. 3a), specificity, 0.75 (95% CI: 0.69-0.81) (Fig. 3b), AUC was 0.87 (95% CI: 0.83-0.89) (Fig. 3c), NLR, 0.21 (95% CI: 0.16-0.29) (Fig. S1a), PLR, 3.42 (95% CI: 2.68-4.35) (Fig. S1b) and DOR, 15.99 (95% CI: 9.89-25.83) (Fig. S1c) (Table 2). The results manifested that circulating microRNAs had a high diagnostic accuracy.

### Diagnostic value of AFP in HBV-related HCC patients

In all of these studies, 9 studies analyzed the diagnostic value of AFP in HBV-related HCC patients. Meta-analysis was taken by using a random effect model (I2 > 50%). The pooled results were displayed as follow: sensitivity, 0.68 (95% CI: 0.62-0.73), specificity, 0.76 (95% CI: 0.61-0.86) , PLR, 2.78 (95% CI: 1.69-4.56), NLR, 0.43 (95% CI: 0.35-0.52), DOR, 6.53 (95% CI: 3.37-12.65) and AUC was 0.73 (95% CI: 0.69-0.77) (Figure S2). The results showed that circulating microRNAs had a better diagnostic accuracy than AFP.

### Diagnostic value of miR-125b in HBV-related HCC patients

**Figure 4:**
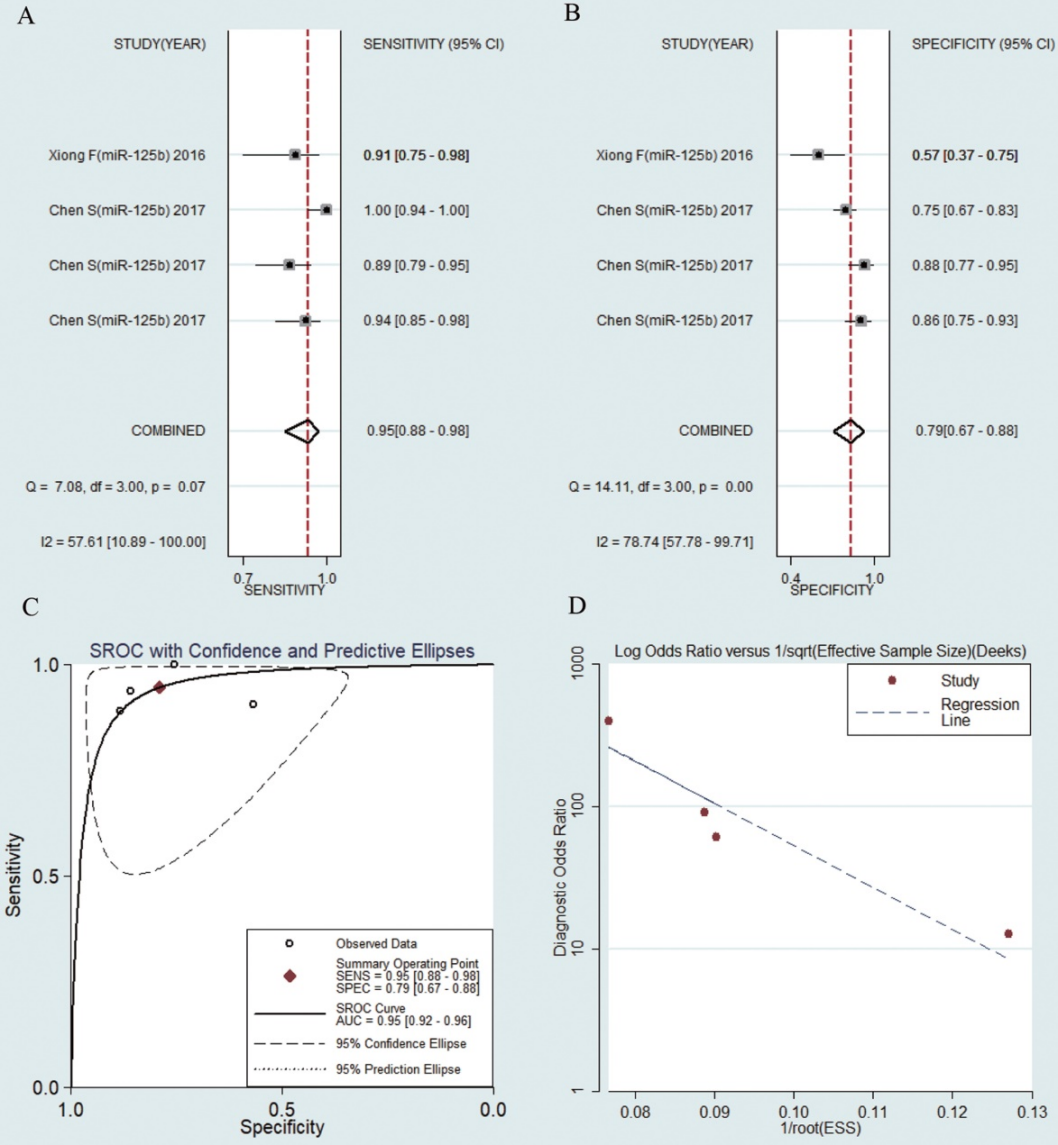
Forest plots of sensitivity, specificity, area under the curve (AUC) and funnel plot of circulating miR-125b for diagnosing HBV-related HCC among 4 studies. (A) Sensitivity; (B) Specificity; (C) AUC; (D) Funnel plot.

MiR-125b were reported in 4 studies of collected researches. The pooled sensitivity was 0.95 (95% CI: 0.88-0.98) (Fig. 4a). The pooled specificity was 0.79 (95% CI: 0.67-0.88) (Fig. 4b), AUC was 0.95 (95% CI: 0.92-0.96) (Fig. 4c), the pooled NLR was 0.07 (95% CI: 0.03-0.16) (Fig. S3a), the pooled PLR was 4.50 (95% CI: 2.76-7.33) (Fig. S3b) and the pooled DOR was 65.58 (95% CI: 24.33-176.77) (Fig. S3c).

### Meta-regression analysis

To find probable sources of heterogeneity, we used logOR as the dependent variable. Regulation mode, miRNA profiling, sample size, internal reference types, specimen types, ethnicity and control groups were considered as covariates (Table 2). The result of I-squared-res value was 66.64%, manifesting the heterogeneity could be explained by 66.64% of the residual variation. The adjusted R-squared was 47.42%, which could explain the variation among the studies; this variation, might be related to the regulation mode (P = 0.016), miRNA profiling (P = 0.026), CHB control group (P = 0.043) and LC control group (P = 0.021). Besides, the variation was not related to sample size (P = 0.276), internal reference types (P = 0.194), specimen types (P = 0.532), ethnicity (P = 0.308) and CHB and LC control group (P = 0.257). Given that, we conducted subgroup analyses.

### Subgroup analysis

Subgroup analyses were conducted according to regulation mode, source of control, miRNA profiling, sample size, internal reference types, and specimen types. The pooled sensitivity, specificity, PLR, NLR, DOR and AUC for each subgroup analysis were listed in Table 3. We found that down-regulated miRNAs assays had a better diagnostic value than up-regulated miRNAs assays in the diagnosis of HBV-HCC: sensitivity (0.91 vs. 0.79), specificity (0.82 vs. 0.72), PLR (4.95 vs. 2.81), NLR (0.11 vs. 0.29), DOR (45.13 vs. 9.59) and AUC (0.94 vs. 0.82). Besides, the assay using multiple miRNAs exhibited a better diagnostic value than single miRNA: sensitivity (0.89 vs. 0.84), specificity (0.84 vs. 0.71), PLR (7.41 vs. 2.94), NLR (0.18 vs. 0.22), DOR (40.22 vs. 13.12) and AUC (0.92 vs. 0.83). Apart from that, the studies with sample size more than 100 were significantly greater than the studies with sample size less than 100 in the diagnosis of HCC in HBV patients: sensitivity (0.84 vs. 0.79), specificity (0.77 vs. 0.65), PLR (3.70 vs. 2.28), NLR (0.20 vs. 0.33), DOR (25.90 vs. 6.96) and AUC (0.88 vs. 0.76). In addition, Serum types had also a higher diagnostic value than plasma types: sensitivity (0.86 vs. 0.65), specificity (0.74 vs. 0.82), PLR (3.32 vs. 3.69), NLR (0.19 vs. 0.43), DOR (17.46 vs. 8.61) and AUC (0.88 vs. 0.81). Internal reference types in qRT-PCR had no influence on the diagnosis (Table 3).

**Figure 5:**
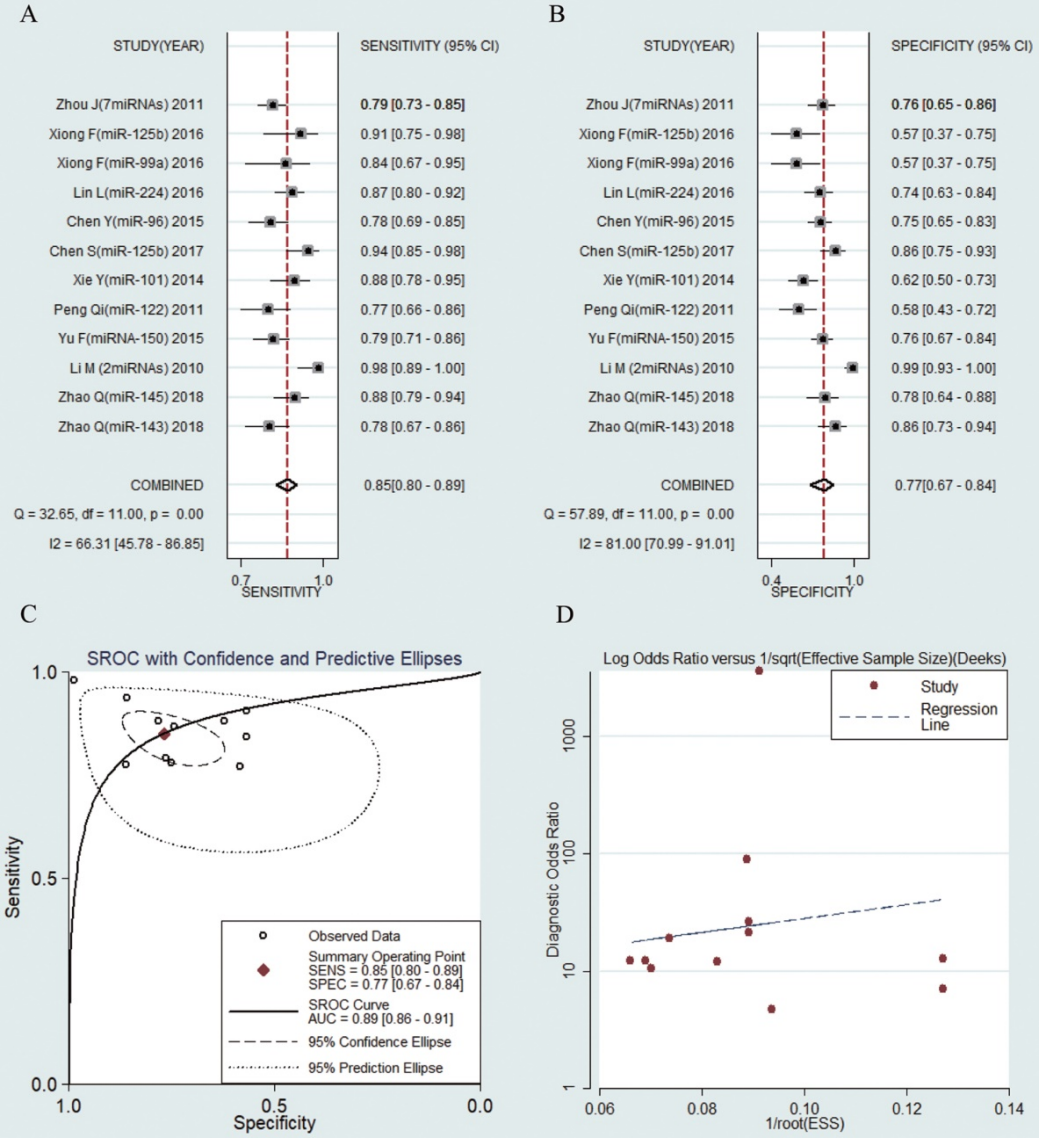
Forest plots of sensitivity, specificity, area under the curve (AUC) and funnel plot of circulating miRNAs for diagnosing HBV-related HCC in patients with chronic hepatitis B among 12 studies. (A) Sensitivity; (B) Specificity; (C) AUC; (D) Funnel plot.

12 studies including 1027 HBV-related HCC patients and 782 CHB patients were analyzed separately. The pooled results were displayed as follows: sensitivity, 0.85 (95% CI: 0.80-0.89) (Fig. 5a), specificity, 0.77 (95% CI: 0.67-0.84) (Fig. 5b), AUC was 0.89 (95% CI: 0.86-0.91) (Fig. 5c), NLR, 0.19 (95% CI: 0.13-0.28) (Fig. S4a), PLR, 3.66 (95% CI: 2.46-5.45) (Fig. S4b) and DOR, 18.85 (95% CI: 9.09-39.10) (Fig. S4c). The results showed that circulating microRNAs had a great diagnostic accuracy for HCC in CHB patients.

**Figure 6:**
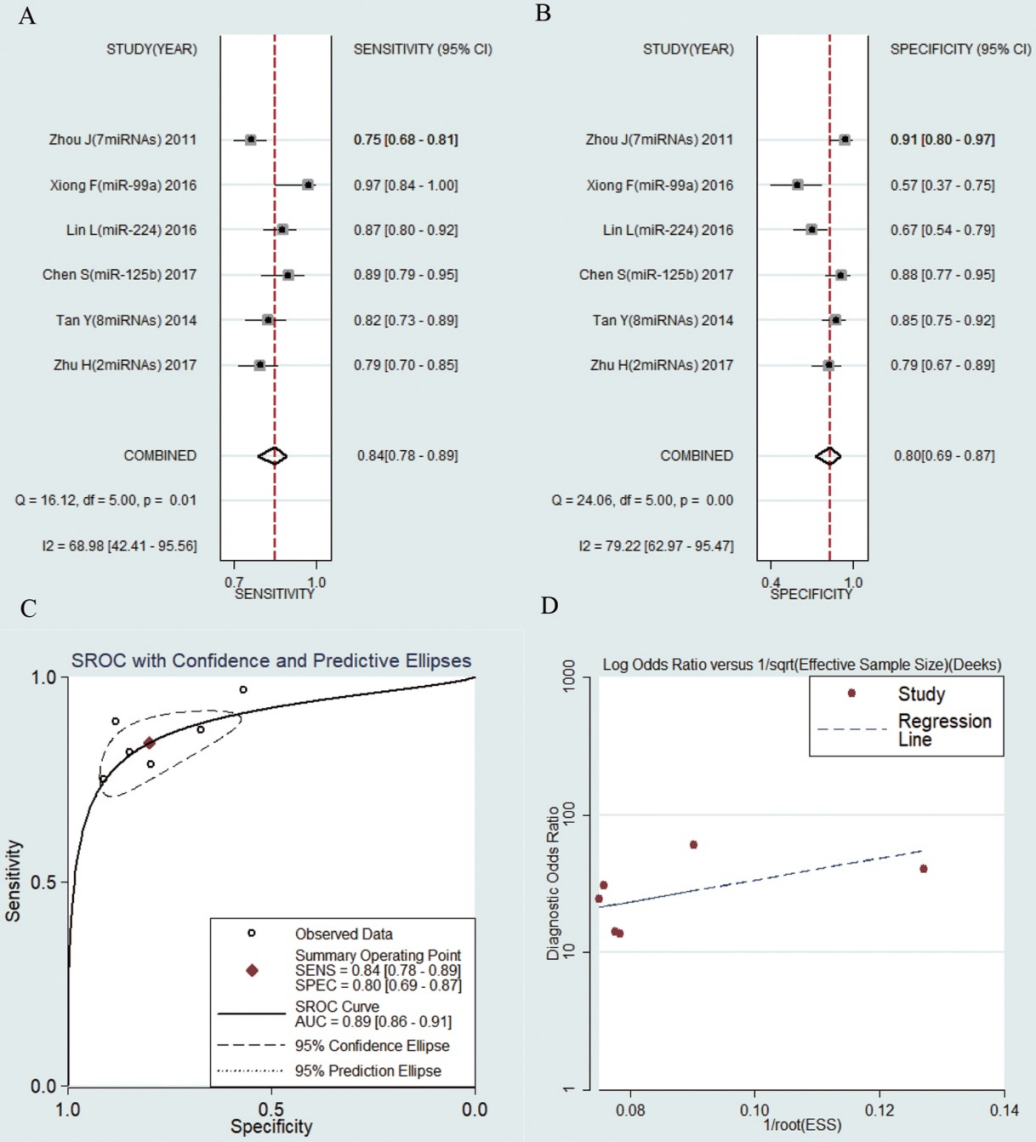
Forest plots of sensitivity, specificity, area under the curve (AUC) and funnel plot of circulating miRNAs for diagnosing HBV-related HCC in patients with liver cirrhosis among 6 studies. (A) Sensitivity; (B) Specificity; (C) AUC; (D) Funnel plot.

6 studies including 638 HBV-related HCC patients and 347 LC patients were analyzed separately. The pooled results were displayed as follows: sensitivity, 0.84 (95% CI: 0.78-0.89) (Fig. 6a), specificity, 0.80 (95% CI: 0.69-0.87) (Fig. 6b), AUC was 0.89 (95% CI: 0.86-0.91) (Fig. 6c), NLR, 0.20 (95% CI: 0.16-0.27) (Fig. S5a), PLR, 4.16 (95% CI: 2.75-6.27) (Fig. S5b) and DOR, 20.47 (95% CI: 13.52-30.98) (Fig. S5c). The results manifested that circulating microRNAs also had a high diagnostic accuracy for HCC in LC patients.

### Publication bias

The publication bias of the included studies was checked by Deeks' funnel plot test. The pooled Deeks' test result of all studies was t = -0.56, P = 0.582 (Fig. 3d), which demonstrated no significant publication bias in this analysis. In additional, when CHB individuals were used as controls, the pooled Deeks' test result was t = 0.45, P = 0. 661 (Fig. 4d), and when LC individuals were used as controls, the pooled Deeks' test result was t = 1.07, P = 0.344 (Fig. 5d), which all indicating no publication bias. As for studies conducting index of AFP, the pooled Deeks' test result was t = 3.74, P = 0. 0007 (Fig. 6d), showing significant publication bias.

### Clinical utility of index test

Fagan's Nomogram is used for calculating post-test probabilities. As was shown in Figure S1d, it was found that when the pre-test probability was set at 20%, the post-test probability arrived at 46% accompanied by a PLR of 3, and the post-test probability arrived at 5% accompanied by a NLR of 0.21.

## Discussions

Hepatits B virus infection remains an outstanding problem and the leading risk factor for hepatocellular carcinoma. It is known that HCC is the leading cause of cancer-associated death and most of patients are diagnosed at advanced stage upon disease discovery. As it would be too late for the optimal treatment when the physiological consequences of cancer were observed, it is crucial for the early detection of HCC to improve patient survival 31. Liver biopsy, the gold procedure for the diagnosis and stage assessment of HCC, is difficult to implement for various complications and impractical to perform frequently. Imaging technologies, such as ultrosound and computed tomography, are limited in identify the severity degree of inflammation and hepatocellular injury. Fortunately, with the increasing development of sequencing technologies and tested approches about liver diseases, a growing number of novel tools with diagnostic function are emerging. Liquid biopsy, which is defined as non-invasive reliable biomarkers, is becoming a hot spot of recently researches. Circulating extracellular vesicles, Cell-free DNA (cfDNA) , Cell-free non-coding RNA (cfRNA) and tumour cells are the promising and potential liquid biopsy possibles as they are performed with satisfying diagnostic and prognostic value^32^. Significantly, microRNAs, the most studied types of cfRNA, have already been confirmed that play a key role in numerous cell activities of hepatocytes. Circulating microRNAs have been widely and continuously studied in HCC in recent years and multiple researches have shown the high sensitivity and specificity of microRNAs in distinguishing HBV-HCC from HBV patients without HCC. To evaluate the practicability and utility of microRNAs as a circulating biomarker in HBV-HCC, we conducted this meta-analysis to provide a comprehensive and up-to-date analysis.

Totally, we included 25 studies from 15 articles with 3841 subjects (2290 HBV-related HCC patients and 1551 HBV patients without HCC) and each study showed acceptable or satisfied sensitivity and specificity. In this meta-analysis, the pooled sensitivity was 0.84 (95% CI: 0.79-0.88) and the pooled specificity was 0.75 (95% CI: 0.69-0.81), showing a significant diagnostic effect in HBV-HCC patients. We also drew the SROC curve and obtained the corresponding AUC to assess the overall diagnostic accuracy, with the ideal result of 0.87 in AUC value, meaning that microRNAs reached the moderate level and almost the high level of evaluation criteria in diagnosis 33, and showing a better diagnostic performance than AFP (AUC, 0.73). We also used the PLR, NLR and DOR to further test the discrimination ability of microRNAs, which can provide more meaningful references for clinical usage. In our meta-analysis, the total DOR, the pooled PLR and NLR were 15.99, 3.42 and 0.41 respectively, indicating that the chance of a correct diagnosis of HBV-HCC individuals was 16 times higher than a false-negative diagnosis of non-HCC but HBV infected patients. However, the PLR is lower than 10 and the NLR is not less than 0.1, which did not reach the general criterion in ruling in or ruling out decision 34.

In this study, we conducted a meta-regression to detect the effection of the regulation mode, miRNA profiling, sample size, internal reference types, specimen types, ethnicity and control groups. The result revealed that regulation mode , miRNA profiling, CHB control group and LC control group were as potential sources of heterogeneity. Furthermore, we measured a subgroup analysis and interestingly, we found that down-regulated miRNAs assays have a better diagnostic value than up-regulated miRNAs assays (DOR, 45.13 vs. 9.59; AUC, 0.94 vs. 0.82), the assay using multiple microRNA showed better diagnostic value than single miRNA (DOR, 40.2 vs 13.1; AUC, 0.92 vs. 0.83), bigger sample size exhibited better diagnostic value than sample size less than 100 (DOR, 25.90 vs. 6.96; AUC, 0.88 vs. 0.76), serum type had better diagnostic value than plasma type (DOR, 17.46 vs. 8.61; AUC, 0.88 vs. 0.81 ). Given that, we may draw a conclusion that down-regulated microRNA panel in serum type and test in bigger size sample can perform the best diagnostic function from HBV-HCC. Curiously, it has been reported that plasma perhaps retain more proteins for co-fractionating miRNAs and should have more clinical applications which were contrary to our study 35, more studies are needed to define which circulating type is the best biological marker sample. Moreover, no obvious diagnostic difference was found in CHB and LC groups and concequently, it may conclude that circulating miRNAs were of high value to diagnose HBV-related HCC in different stages of HBV infected patients.

In our study, the downregulated circulating miR-125b showed a significant diagnostic achievement. It has already been reported that downregulation of miR-125b were associated with cell proliferation by enabling the hepatocytes to exist under conditions of low nutrition and therapy of chemotherapy 36. More specifically, miR-125b can inhibit the tumorigenesis through targeting Mcl-1 and IL-6R, which acted important roles in apoptosis and immune response 36-38. In the genic aspect, oncogene LIN28B were directly targeted by miR-125b and ultimately, suppressed HCC cellular differentiation and metastasis 37-39. Bcl-2, an anti-apoptotic gene, can affect hepatocarcinogenesis and chemoresistance as its subsequent up-regulation of expression 38. It is noteworthy that a recent study showed the therapeutic value of man-made miR-125b mimics through decreasing the target molecule of cancer stem cell (CSC) using the HCC xenograft model in mice 40 and it may provide a promising way to cure HCC.

This meta-analysis may have some limitations: (1) although we performed the extensive literature search, some related studies may still omitted and not included in this meta-analysis. (2) research sizes were relatively small and consequently, our findings need to be further confirmed. (3) due to researches' limited data and different criteria, we did not extract cut-off values, different cut-off values may result in the inconsistent conclusions. (4) all of the included studied were from Asia and mostly from China.

In conclusion, despite these deficiencies, our meta-analysis demonstrated that microRNAs could distinguish the HBV-related HCC from HBV patients without HCC with high sensitivity and specificity and may serve as promising circulating biomarkers in the early diagnosis in HBV-HCC.

## Supplementary Material

Supplementary figures and tables.

## Figures and Tables

**Table 1:** Characteristics of the included studies

First author	Year	Country	microRNAs	Regulation mode	Sample size				Specimen	Diagnostic power		
					Case	Number	Control	Number		Sen (%)	Spe (%)	AUC
Zhu H	2017	China	miRNA clusters	Upregulated	HCC	121	LC	63	Serum	0.785	0.793	0.859
Zhao Q	2018	China	miR-143	Downregulated	HCC	85	CHB	50	Serum	0.776	0.860	0.813
Zhao Q	2018	China	miR-145	Downregulated	HCC	85	CHB	50	Serum	0.882	0.780	0.852
Li M	2010	China	miRNA clusters	Downregulated	HCC	50	CHB	75	Serum	0.985	0.985	0.992
Tan Y	2014	China	miRNA clusters	Upregulated	HCC	103	LC	78	Serum	0.816	0.846	0.892
Yu F	2015	China	miRNA-150	Downregulated	HCC	120	CHB	110	Serum	0.791	0.765	0.881
Amit Ghosh	2016	Indian	miR-126	Upregulated	HCC	49	CHB+LC	38	Plasma	0.630	0.580	0.671
Amit Ghosh	2016	Indian	miR-142-3p	Upregulated	HCC	49	CHB+LC	38	Plasma	0.320	0.910	0.553
Peng Qi	2011	China	miR-122	Upregulated	HCC	70	CHB	48	Serum	0.776	0.578	0.630
Xie Y	2014	China	miR-101	Downregulated	HCC	67	CHB	79	Serum	0.881	0.620	0.777
Chen S	2017	China	miR-125b	Downregulated	HCC	64	CHB	63	Serum	0.938	0.857	0.958
Chen S	2017	China	miR-125b	Downregulated	HCC	64	LC	59	Serum	0.891	0.881	0.958
Chen S	2017	China	miR-125b	Downregulated	HCC	64	CHB+LC	122	Serum	1.000	0.755	0.943
Chen Y	2015	China	miR-96	Upregulated	HCC	104	CHB	100	Serum	0.779	0.753	0.803
Hung C	2016	China	miR-122	Upregulated	HCC	120	DN	30	Serum	0.667	0.567	0.648
Hung C	2016	China	let-7b	Upregulated	HCC	120	DN	30	Serum	0.825	0.467	0.633
Lin L	2016	China	miR-224	Upregulated	HCC	122	CHB+LC	135	Serum	0.865	0.711	0.840
Lin L	2016	China	miR-224	Upregulated	HCC	122	LC	61	Serum	0.865	0.667	0.832
Lin L	2016	China	miR-224	Upregulated	HCC	122	CHB	74	Serum	0.865	0.745	0.846
Li L	2012	China	miR-18a	Upregulated	HCC	101	CHB+LC	30	Serum	0.772	0.700	0.775
Xiong F	2016	China	miR-99a	Upregulated	HCC	32	CHB	30	Serum	0.844	0.567	0.694
Xiong F	2016	China	miR-125b	Downregulated	HCC	32	CHB	30	Serum	0.906	0.567	0.703
Xiong F	2016	China	miR-99a	Upregulated	HCC	32	LC	30	Serum	0.967	0.563	0.696
Zhou J	2011	China	miRNA clusters	Upregulated	HCC	196	CHB	72	Plasma	0.791	0.764	0.842
Zhou J	2011	China	miRNA clusters	Upregulated	HCC	196	LC	56	Plasma	0.750	0.911	0.884

HCC: Hepatocellular carcinoma, LC: Liver cirrhosis, CHB: Chronic hepatitis B, DN: Dysplastic nodule, Sen: Sensitivity, Spe: Specificity, AUC: area under the curve.

**Table 2:** The meta-regression analysis in the binary classification of variable data using the odds ratio (OR)

LogOR	Coef.	Std. Err.	t	P>|t|	[95% Conf. Interval]	
Regulation mode	-1.302928	0.492039	-2.65	0.016	-2.336663	-0.2691922
miRNA profiling	1.869125	0.7728873	2.42	0.026	0.2453491	3.492901
Sample size	-0.9457466	0.841248	-1.12	0.276	-2.713143	0.8216499
Internal reference types	0.7083823	0.525466	1.35	0.194	-0.3955807	1.812345
Specimen types	0.5501442	0.8642539	0.64	0.532	-1.265586	2.365874
Ethnicity	1.472494	1.404431	1.05	0.308	-1.478106	4.423094
Control-CHB	1.596359	0.7393835	2.16	0.043	0.0587269	3.133991
Control-LC	1.99139	0.7978941	2.50	0.021	0.332078	3.650701
Control-CHB+LC	0.9694282	0.8318447	1.17	0.257	-0.7604876	2.699344

LogOR was used as response variables as well as regulation modes, miRNA profiling, sample size, internal reference types, specimen types, ethnicity, and source of controls group were as covariates. Estimate of between-study variance tau2 = 0.6265. Residual variation due to heterogeneity: I-squared_res = 66.64%. Proportion of between-study variance explained: Adj R-squared = 47.42%. Joint test for all covariates with Knapp-Hartung modifcation: Prob > F = 0.0712.

**Table 3:** Summary estimates of diagnostic power and their 95% confidence intervals

Subgroup		Sensitivity (95% CI)	Specificity (95% CI)	Positive LR (95% CI)	Negative LR (95% CI)	DOR (95% CI)	AUC (95% CI)
**Regulation mode**							
Upregulated	16	0.79 [0.73-0.84]	0.72 [0.65-0.78]	2.81 [2.26-3.50]	0.29 [0.23-0.38]	9.59 [6.62-13.88]	0.82 [0.78-0.85]
Downregulated	9	0.91 [0.84-0.95]	0.82 [0.71-0.89]	4.95 [2.98-8.22]	0.11 [0.06-0.20]	45.13 [16.81-121.14]	0.94 [0.91-0.95]
**Source of control**							
Chronic hepatitis B	12	0.85 [0.80-0.89]	0.77 [0.67-0.84]	3.66 [2.46-5.45]	0.19 [0.13-0.28]	18.85 [9.09-39.10]	0.89 [0.86-0.91]
Liver cirrhosis	6	0.84 [0.78-0.89]	0.80 [0.69-0.87]	4.16 [2.75-6.27]	0.20 [0.16-0.27]	20.47 [13.52-30.98]	0.89 [0.86-0.91]
**miRNA profiling**							
Multiple miRNAs	5	0.89 [0.75-0.95]	0.84 [0.72-0.91]	7.41 [2.90-18.94]	0.18 [0.10-0.36]	40.22 [8.63-187.51]	0.92 [0.90-0.94]
Single miRNA	20	0.84 [0.78-0.89]	0.71 [0.66-0.77]	2.94 [2.42-3.57]	0.22 [0.16-0.32]	13.12 [8.18-21.05]	0.83 [0.79-0.86]
**Sample size**							
**≥**100	20	0.84 [0.80-0.88]	0.77 [0.71-0.82]	3.70 [2.82-4.86]	0.20 [0.15-0.27]	25.90 [11.07-30.46]	0.88 [0.85-0.91]
<100	5	0.79 [0.53-0.92]	0.65 [0.49-0.79]	2.28 [1.67-3.11]	0.33 [0.15-0.72]	6.96 [2.98-16.27]	0.76 [0.72-0.79]
**Internal reference types in qRT-PCR**							
U6	13	0.83 [0.73-0.90]	0.75 [0.68-0.81]	3.32 [2.49-4.43]	0.22 [0.13-0.37]	14.90 [7.41-29.96]	0.84 [0.81-0.87]
Non-U6	12	0.83 [0.78-0.87]	0.76 [0.66-0.84]	3.49 [2.33-5.24]	0.22 [0.16-0.30]	15.74 [8.29-29.90]	0.87 [0.84-0.90]
**Specimen types**							
Serum	21	0.86 [0.82-0.89]	0.74 [0.68-0.80]	3.32 [2.57-4.29]	0.19 [0.14-0.26]	17.46 [10.44-29.19]	0.88 [0.85-0.91]
Plasma	4	0.65 [0.46-0.80]	0.82 [0.66-0.92]	3.69 [1.83-7.43]	0.43 [0.27-0.69]	8.61 [3.24-22.86]	0.81 [0.77-0.84]
Total	25	0.84 [0.79-0.88]	0.75 [0.69-0.81]	3.42 [2.68-4.35]	0.21 [0.16-0.29]	15.99 [9.89-25.83]	0.87 [0.83-0.89]

LR: likelihood ratio, DOR: diagnostic odds ratio, AUC: area under the curve, CI: confidence interval.

## Abbreviations

miRNAs: MicroRNAs
SEN: Sensitivity
SPE: Specificity
PLR: Positive likelihood ratio
NLR: Negative likelihood ratio
SROC: Summary receiver- operating characteristics
AUC: Area under the Curve of ROC
DOR: Diagnostic odds ratio
CI: confidence interval
qRT-PCR: Quantitative reverse transcription polymerase chain reaction
HCC: Hepatocellular carcinoma
LC: Liver cirrhosis
CHB: Chronic hepatitis B
AFP: Alpha fetoprotein
