# Supplementary material for: Diagnostic Value of Circulating microRNAs in Hepatitis B Virus-Related Hepatocellular Carcinoma: A Systematic Review and Meta-Analysis
Source: J Cancer. 2019 Aug 20;10(20):4754–64. doi: 10.7150/jca.32833 (PMC6775527; doi:10.7150/jca.32833)
Supplement: Supplementary file 1 — Supplementary figures and tables. [file jcav10p4754s1.pdf]

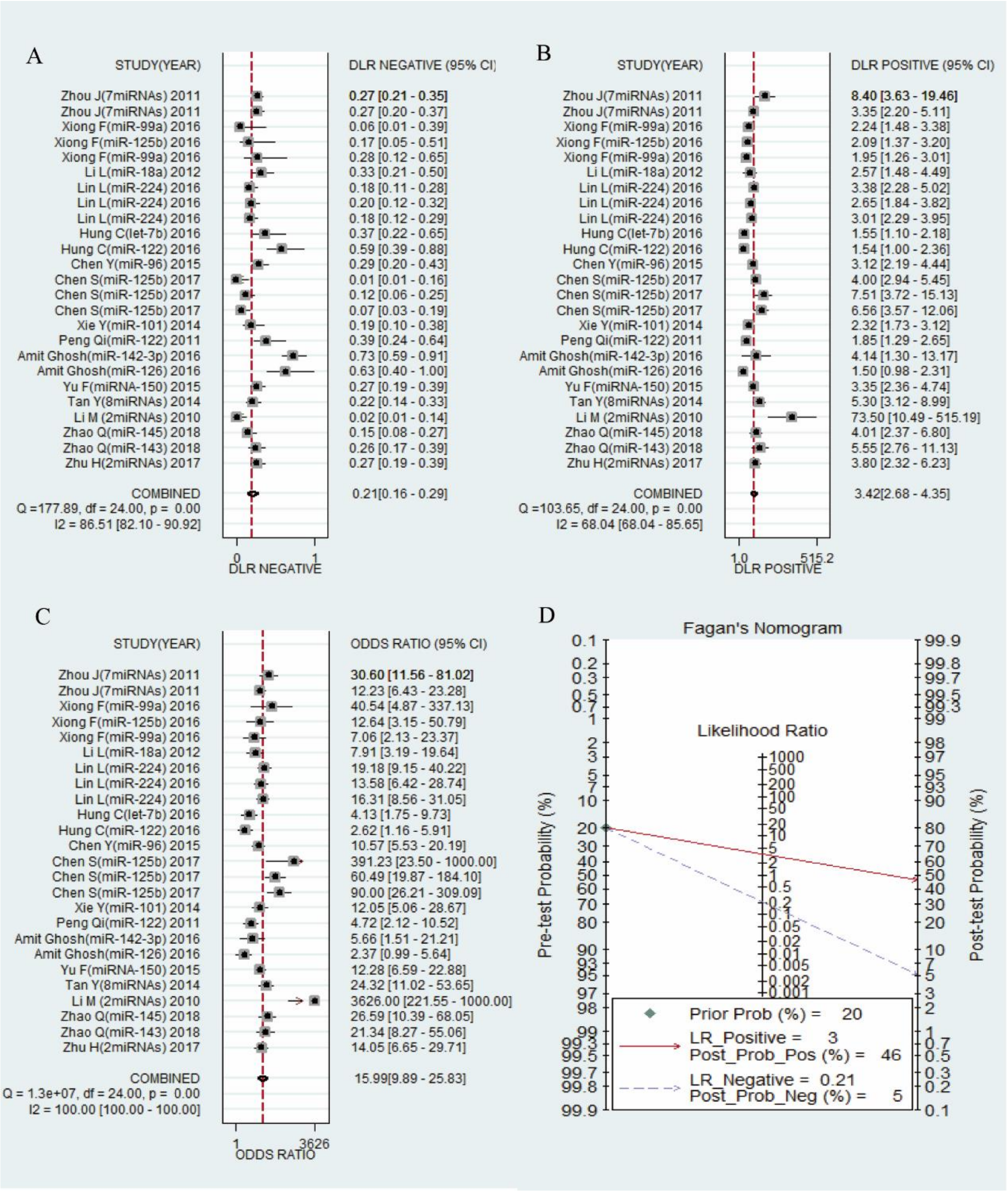

Figure S1: Forest plots of negative likelihood ratio (NLR), positive likelihood ratio (PLR), diagnostic odds ratio (DOR) and Fagan's nomogram of circulating miRNAs for diagnosing HBV-related HCC among 25 studies. (A) NLR; (B) PLR; (C) DOR; (D) Fagan's nomogram.

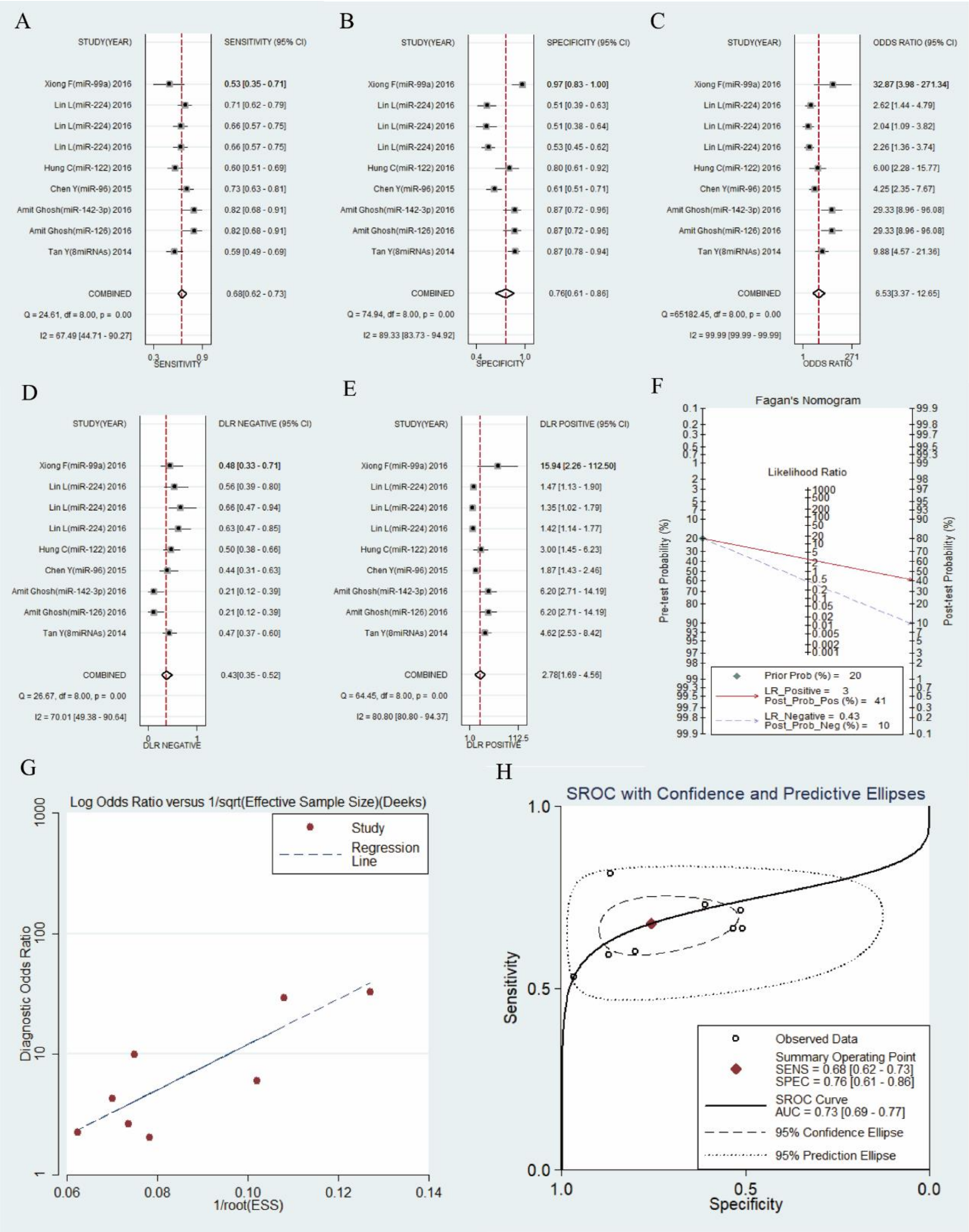

Figure S2: Forest plots of sensitivity, specificity, diagnostic odds ratio (DOR), negative likelihood ratio (NLR), positive likelihood ratio (PLR), Fagan's nomogram, funnel plot and area under the curve (AUC) of AFP for diagnosing HBV-related HCC among 9 studies. (A) Sensitivity; (B) Specificity; (C) DOR; (D) NLR; (E) PLR; (F) Fagan's nomogram; (G) Funnel plot; and (H) AUC.

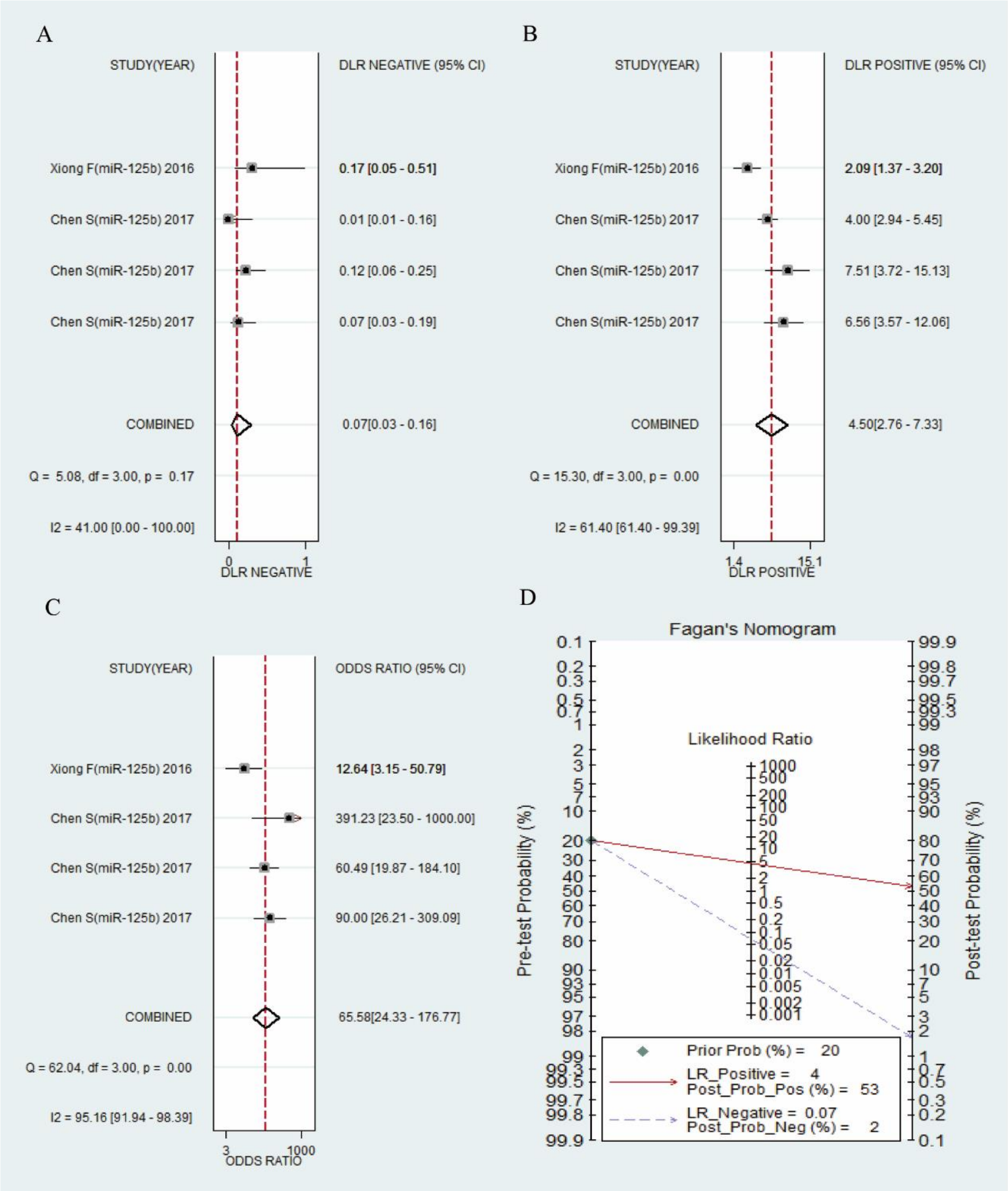

Figure S3: Forest plots of negative likelihood ratio (NLR), positive likelihood ratio (PLR), diagnostic odds ratio (DOR) and Fagan's nomogram of circulating miR-125b for diagnosing HBV-related HCC among 4 studies. (A) NLR; (B) PLR; (C) DOR; (D) Fagan's nomogram.

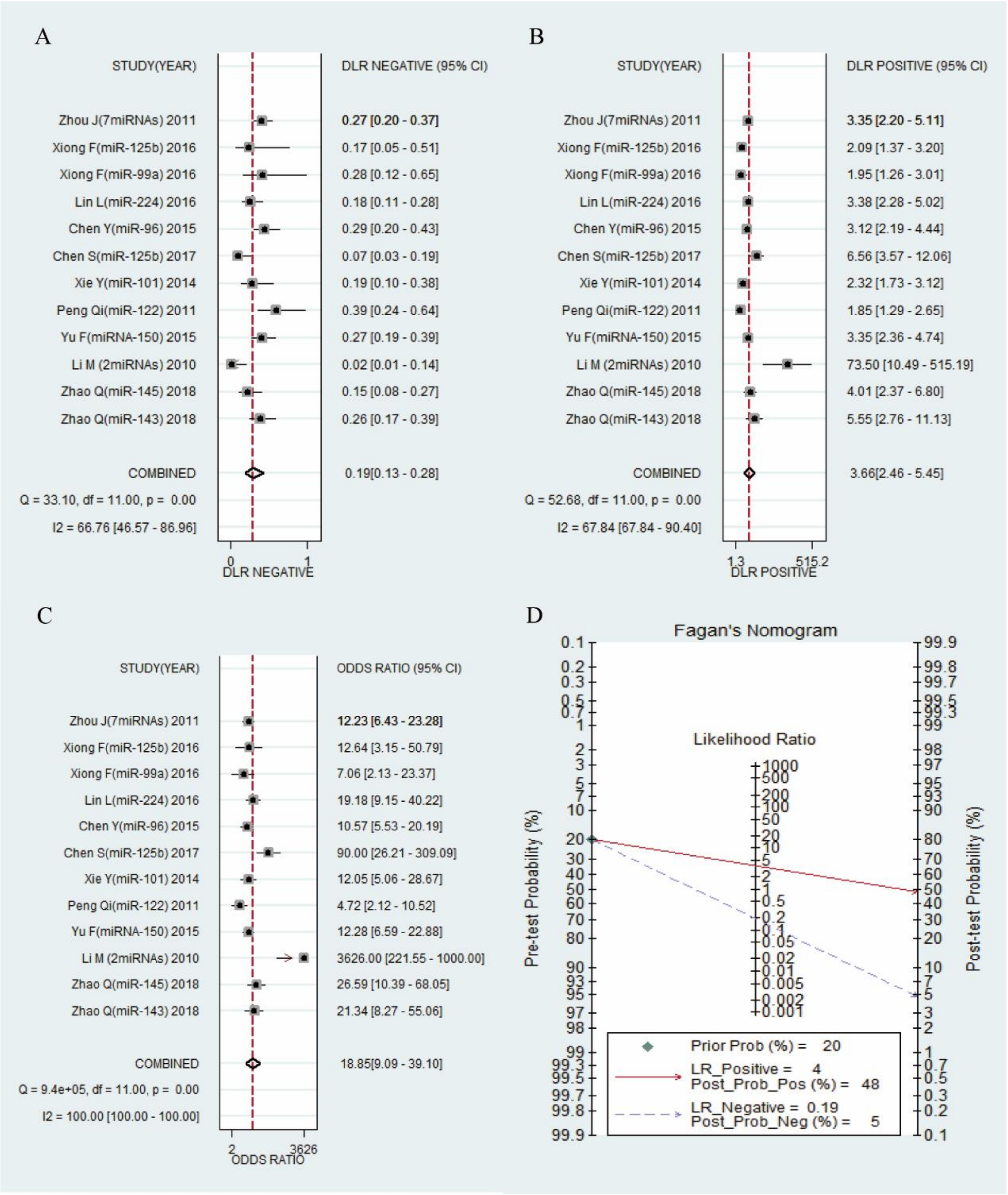

A
STUDY(YEAR)
DLR NEGATIVE (95% CI)
Zhou J(7miRNAs) 2011 0.27 [0.20 - 0.37]
Xiong F(miR-125b) 2016 0.17 [0.05 - 0.51]
Xiong F(miR-99a) 2016 0.28 [0.12 - 0.65]
Lin L(miR-224) 2016 0.18 [0.11 - 0.28]
Chen Y(miR-96) 2015 0.29 [0.20 - 0.43]
Chen S(miR-125b) 2017 0.07 [0.03 - 0.19]
Xie Y(miR-101) 2014 0.19 [0.10 - 0.38]
Peng Qi(miR-122) 2011 0.39 [0.24 - 0.64]
Yu F(miRNA-150) 2015 0.27 [0.19 - 0.39]
Li M (2miRNAs) 2010 0.02 [0.01 - 0.14]
Zhao Q(miR-145) 2018 0.15 [0.08 - 0.27]
Zhao Q(miR-143) 2018 0.26 [0.17 - 0.39]
COMBINED 0.19[0.13 - 0.28]
Q = 33.10, df = 11.00, p = 0.00
I2 = 66.76 [46.57 - 86.96]
0 1
DLR NEGATIVE
B
STUDY(YEAR)
DLR POSITIVE (95% CI)
Zhou J(7miRNAs) 2011 3.35 [2.20 - 5.11]
Xiong F(miR-125b) 2016 2.09 [1.37 - 3.20]
Xiong F(miR-99a) 2016 1.95 [1.26 - 3.01]
Lin L(miR-224) 2016 3.38 [2.28 - 5.02]
Chen Y(miR-96) 2015 3.12 [2.19 - 4.44]
Chen S(miR-125b) 2017 6.56 [3.57 - 12.06]
Xie Y(miR-101) 2014 2.32 [1.73 - 3.12]
Peng Qi(miR-122) 2011 1.85 [1.29 - 2.65]
Yu F(miRNA-150) 2015 3.35 [2.36 - 4.74]
Li M (2miRNAs) 2010 73.50 [10.49 - 515.19]
Zhao Q(miR-145) 2018 4.01 [2.37 - 6.80]
Zhao Q(miR-143) 2018 5.55 [2.76 - 11.13]
COMBINED 3.66[2.46 - 5.45]
Q = 52.68, df = 11.00, p = 0.00
I2 = 67.84 [67.84 - 90.40]
1.3 515.2
DLR POSITIVE
C
STUDY(YEAR)
ODDS RATIO (95% CI)
Zhou J(7miRNAs) 2011 12.23 [6.43 - 23.28]
Xiong F(miR-125b) 2016 12.64 [3.15 - 50.79]
Xiong F(miR-99a) 2016 7.06 [2.13 - 23.37]
Lin L(miR-224) 2016 19.18 [9.15 - 40.22]
Chen Y(miR-96) 2015 10.57 [5.53 - 20.19]
Chen S(miR-125b) 2017 90.00 [26.21 - 309.09]
Xie Y(miR-101) 2014 12.05 [5.06 - 28.67]
Peng Qi(miR-122) 2011 4.72 [2.12 - 10.52]
Yu F(miRNA-150) 2015 12.28 [6.59 - 22.88]
Li M (2miRNAs) 2010 3626.00 [221.55 - 1000.00]
Zhao Q(miR-145) 2018 26.59 [10.39 - 68.05]
Zhao Q(miR-143) 2018 21.34 [8.27 - 55.06]
COMBINED 18.85[9.09 - 39.10]
Q = 9.4e+05, df = 11.00, p = 0.00
I2 = 100.00 [100.00 - 100.00]
2 3626
ODDS RATIO
D
Fagan's Nomogram
Likelihood Ratio
Pre-test Probability (%)
Post-test Probability (%)
Prior Prob (%) = 20
LR_Positive = 4
Post_Prob_Pos (%) = 48
LR_Negative = 0.19
Post_Prob_Neg (%) = 5

Figure S4: Forest plots of negative likelihood ratio (NLR), positive likelihood ratio (PLR), diagnostic odds ratio (DOR) and Fagan's nomogram of circulating miRNAs for diagnosing HBV-related HCC in patients with chronic hepatitis B among 12 studies. (A) NLR; (B) PLR; (C) DOR; (D) Fagan's nomogram.

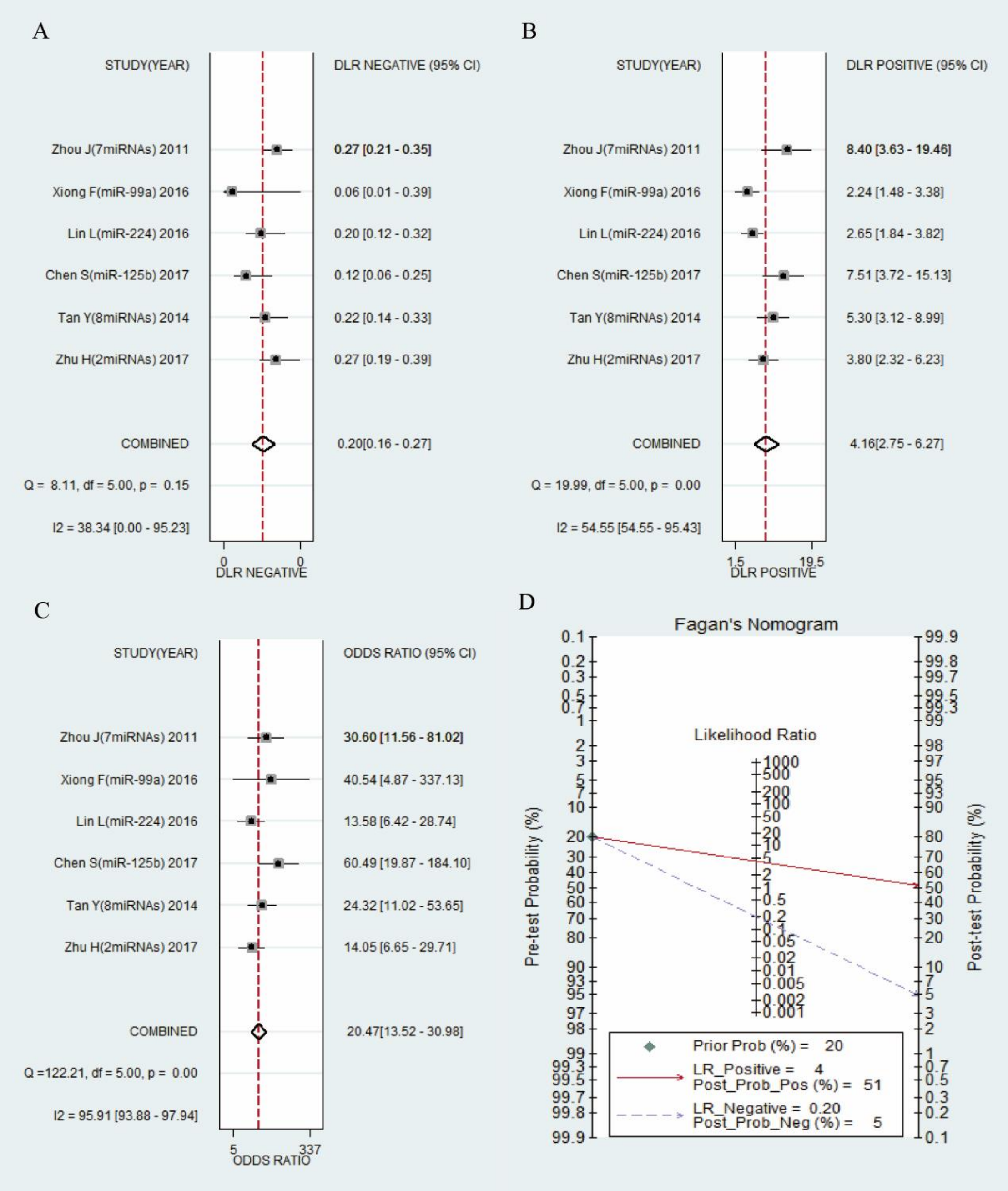

Figure S5: Forest plots of negative likelihood ratio (NLR), positive likelihood ratio (PLR), diagnostic odds ratio (DOR) and Fagan's nomogram of circulating miRNAs

for diagnosing HBV-related HCC in patients with liver cirrhosis among 6 studies. (A) NLR; (B) PLR; (C) DOR; (D) Fagan's nomogram.
